# Children of the Future Age

**Published:** 1988-11

**Authors:** J. K. Lloyd

**Affiliations:** The Institute if Child Health and Hospital for Sick Children, Great Ormond Street, London


### Children of the Future Age

**Professor J. K. Lloyd**
The Institute if Child Health and Hospital for Sick Children, Great Ormond Street, London

Professor Lloyd began by quoting from William Blake's Songs of Experience, words written 37 years after the founding of the BRI from which she derived the title of her paper:

*Children of the future age,*
*Reading this indignant page,*
*Know that those who loved you best*
*Were often thwarted by the rest.*

These lines written 200 years ago are still true. In 1976, a group chaired by Professor Donald Court produced in two volumes a report *Fit for the Future*, now always known as the Court Report, this reminded us that childhood is not an incomplete form of the adult state and that children are human beings in their own right. It pleaded for a child- and family-centred service that should be integrated for the whole child, where skilled help was readily available for both the physical and the emotional needs of children and that it should be increasingly orientated towards prevention. Ten years on we have another report, *'Investing in the future'* by the Policy and Practice Review Group of the National Children's Bureau, and chaired by Professor Graham. It looked at what had been achieved during the previous ten years and sadly came to the conclusion that despite some progress it was not possible to view the health and social conditions of many children and the development of the child health services with anything but deep dissatisfaction. The reasons were identified partly with economic decline and diminution of resources that have gone into the health service, but also with interprofessional and even intraprofessional disagreements resulting in divided responsibilities for the care of children.

The children of the future will want to be born without defects, without being damaged in the birth process and to be able to grow up without disease.

Birth defects are assuming an increasing importance as other scourges of children have been conquered. Their prevention involves maternal health, genetic counselling, prenatal diagnosis and even prenatal therapy. Understanding of the effect of maternal ill health on birth defects is increasing; the story of maternal rubella and of the effects of drugs in pregnancy is well known, and we are now moving into the era of looking at micronutrients and vitamin supplementation to prevent neural tube defects. An aspect of maternal health in which colleagues in Professor Lloyd's department had recently been involved related to maternal phenylketonuria and the effect of high levels of amino acids even at conception in producing birth defects. This work showed that dietetic control could eliminate fetal malformations from developing in children at risk.

Genetic counselling also has enormous potential to influence malformations and other speakers were expected to develop this theme.

Prenatal diagnosis can now be made in a very large number of ways, from population screening to diagnosis in individuals, by chorion villus sampling in early pregnancy, by amniocentesis, by ultrasound, by fetoscopy with the ability to obtain blood samples and to take liver biopsies. The increased chances of making a prenatal diagnosis open up possibilities for therapeutic intervention during pregnancy. Much more positive measures than mere termination also become possible with the improved methods of treatment of affected infants. For example treatment is now available for a range of genetic diseases including enzyme induction in neonatal jaundice, enzyme replacement therapy by bone marrow transplantation, coenzyme administration, the induction of alternative pathways in some of the urea cycle defects and substrate restriction as used for phenylketonuria and some of the other amino acidopathies. It may be possible to remove diseased tissue eg retinoblastoma at an early stage if it has been predicted and there are the exciting possibilities of tissue replacement and transplantation.

It is still true that birth is the most dangerous moment of life except the moment of death and the healthy fetus wants to be born without damage during that dangerous period. Birth injury is more likely to occur in the immature infant and more and more of these tiny babies are being rescued by the excellence of perinatal care. Every paediatrician and obstetrician would however like to see immaturity prevented.

Having been born healthy and safely the infant wishes to grow up without disease and much of paediatrics is about disease prevention. The combination of malnutrition and infection kills more children in the world than anything else. Undernutrition may nowadays be rare in so-called developed countries but overnutrition is rampant. Immunisation has prevented a tremendous amount of infectious disease worldwide and a number of conditions have been eliminated. Professor Lloyd recalled that the bell which summoned her to dinner as a resident at the BRI was in former times used to summon help for a case of diphtheria needing tracheostomy. Diphtheria, polio and small pox have gone, but we must do better in eliminating diseases such as measles which has all but disappeared from the N American continent. For the future it is not too wild to even think of immunisation against certain types of childhood cancer.

Finally Health Education has a part to play. Atherosclerosis has its origins in childhood and much adult disease may be prevented by education about healthy eating. Drug addiction, smoking and drinking are starting at younger and younger ages. Accidents are the commonest causes of death in childhood after the age of one year and all these things should be the target of Health Education and the concern of doctors.

A healthy and happy future for today's children requires the vigorous pursuit of both therapeutic and preventive medicine and modern technology is giving us the tools for the job.

